# Literary Notes

**Published:** 1906-09

**Authors:** 


### LITERARY NOTES.

Hon. De Alva Stanwood Alexander, M.C., of Buffalo, is the author of a work entitled "A Practical History of the State of New York," just from the press of Henry Holt and Company. It is an octavo in two volumes of over 400 pages each and contains interesting material relating to the politics of the Empire state from the constitution of 1777 down to the civil war period of 1861. Especially does it record in fascinating manner the rivalries of Hamilton and Clinton and Burr; of Tompkins, of DeWitt Clinton, and of Van Buren. To every person who would familiarise himself with the political history of this interesting and important period,—and who with intelligence would not?,—this work will prove a most acceptable and instructive addition to the library.

Messrs. W. B. Saunders Company announce for publication in the early fall the following works: Keen's Surgery, Its Principles and Practice (Volume I); Sobotta and McMurrich's Human Anatomy (Volume III); Webster's Textbook of Gynecology; Hill's Histology and Organography; McConnell's Pathology; Morrow's Immediate Care of the Injured; Stevenson's Photoscopy (Retinoscopy and Skiascopy); Preiswerk and Warren's Atlas of Dentistry; Goepp's State Board Questions and Answers; Lusk's Elements of Nutrition.

The most important announcement is the new work on surgery, edited by Dr. W. W. Keen, complete in five octavo volumes, and containing over 1500 original illustrations. The entire work is written by the leaders of modern surgery—men whose names are inseparably associated with the subjects upon which they have written. Keen's Surgery is intended to represent the best surgical practice of today.

THE Summer number of *The Quarterly Journal of Inebriety* is a particularly notable issue of this interesting and valuable publication. It has been greatly enlarged and its typographical appearance is exceptionally attractive. Among the leading articles in this number are: The Relation of Alcohol to Tuberculosis, by J. W. Grosvenor; Physiological Action of Tea as a Beverage, by Sir Lauder Brunton; Morbid Predisposing Causes in Dipsomania, by W. L. Howard; Reflexes from the Eye in Narcosomania, by T. H. Evans; The Alcohol Cult, by John Madden; Comparison of the Effects of Alcohol and Opium by W. H. Park; Two articles by the editor, Dr. T. D. Crothers, one on Unrecognised Toxic Insanities, and another on Farmfield Reformatory for Inebriate Women, also appear. Many pages of editorials, abstracts, reviews and comments complete the issue.

